# Hydrazine solution processed Sb_2_S_3_, Sb_2_Se_3_ and Sb_2_(S_1−x_Se_x_)_3_ film: molecular precursor identification, film fabrication and band gap tuning

**DOI:** 10.1038/srep10978

**Published:** 2015-06-04

**Authors:** Bo Yang, Ding-Jiang Xue, Meiying Leng, Jie Zhong, Liang Wang, Huaibing Song, Ying Zhou, Jiang Tang

**Affiliations:** 1 Wuhan National Laboratory for Optoelectronics (WNLO); 2 School of Optical and Electronic Information, Huazhong University of Science and Technology (HUST), Wuhan, 430074, China

## Abstract

Sb_2_(S_1−x_Se_x_)_3_ (0 ≤ x ≤ 1) compounds have been proposed as promising light-absorbing materials for photovoltaic device applications. However, no systematic study on the synthesis and characterization of polycrystalline Sb_2_(S_1−x_Se_x_)_3_ thin films has been reported. Here, using a hydrazine based solution process, single-phase Sb_2_(S_1−x_Se_x_)_3_ films were successfully obtained. Through Raman spectroscopy, we have investigated the dissolution mechanism of Sb in hydrazine: 1) the reaction between Sb and S/Se yields [Sb_4_S_7_]^2-^/[Sb_4_Se_7_]^2-^ ions within their respective solutions; 2) in the Sb-S-Se precursor solutions, Sb, S, and Se were mixed on a molecular level, facilitating the formation of highly uniform polycrystalline Sb_2_(S_1−x_Se_x_)_3_ thin films at a relatively low temperature. UV-vis-NIR transmission spectroscopy revealed that the band gap of Sb_2_(S_1−x_Se_x_)_3_ alloy films had a quadratical relationship with the Se concentration x and it followed the equation 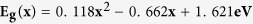, where the bowing parameter was 0.118 eV. Our study provides a valuable guidance for the adjustment and optimization of the band gap in hydrazine solution processed Sb_2_(S_1−x_Se_x_)_3_ alloy films for the future fabrication of improved photovoltaic devices.

Recently, antimony selenide (antimonselite, Sb_2_Se_3_) has been intensively explored as a light-absorbing material for photovoltaic device applications, owing to its simple, low-cost and non-toxic composition, suitable band gap (approximately 1.1 eV) and high optical absorption coefficient of above 10^5^ cm^−1^ at short wavelength^1^,^2^,^3^,^4^,^5^. Within one year, solution processed Sb_2_Se_3_ sensitized solar cells fabricated using a single-source precursor have obtained a power-conversion efficiency (PCE) of 3.21% by Seok’s group^3^, and superstrate Sb_2_Se_3_ solar cells fabricated via thermal evaporation have achieved 3.7% device efficiency by our group^6^, suggesting the promise of Sb_2_Se_3_ for photovoltaic absorber application.

From a device perspective, it would be desirable to introduce S into the Sb_2_Se_3_ absorber film forming alloyed Sb_2_(S_1-x_Se_x_)_3_ (0 ≤ x ≤ 1) film, which could have compositon-dependent properties such as band gap and band position tunability, similar to the Ga alloying in CuInSe_2_ thin film solar cells^7^. In such a way, optimal band alignment and back field grading could be realized, enabling better device performance^8^. Unfortunately, alloyed Sb_2_(S_1-x_Se_x_)_3_ (0 ≤ x ≤ 1) compunds are rarely explored thus far except the following two examples: in 2008, El-Sayad *et al*. reported amorphous Sb_2_(S_1-x_Se_x_)_3_ (x = 0, 1/3, 2/3, and 1) films via thermal evaportion^9^; and in 2009, Deng *et al*. presented the synthesis of Sb_2_(S_1-x_Se_x_)_3_ (0 ≤ x ≤ 1) nanotubes via colloidal synthetic method^10^. To our best knowledge, detailed study of the synthesis and characterization of polycrystalline Sb_2_(S_1-x_Se_x_)_3_ (0 ≤ x ≤ 1) thin films, which is most relevant to photovoltaic application, is missing in the literature.

In this report, we have prepared polycrystalline Sb_2_(S_1-x_Se_x_)_3_ (0 ≤ x ≤ 1) films using hydrazine solution processing. As previously reported, hydrazine is known as a powerful solvent to dissolve many metals in the presence of excess chalcogens without introducing extra C and O impurities^11^,^12^,^13^. After annealing, hydrazine and excess chalcogens are removed, leaving behind a polycrystalline metal chalcogenide film suitable for optoelectronic device applications^14^. For example, ultrathin SnS_x_Se_2-x_ films with mobility greater than 10 cm^2^ V^−1^ s^−1^ and the current champion 12.6% CZTSSe solar cell were all obtained through the hydrazine solution processing^15^,^16^. Furthermore, the composition of final film could be simply tuned by varying S and Se loading in the precursor solution, facilitating the fabrication of Sb_2_(S_1-x_Se_x_)_3_ films with continuously tunable constituents, in analogy to the hydrazine solution processed CuIn(Se,S)_2_ films^17^.

Precise composition control over the Sb_2_(S_1−x_Se_x_)_3_ thin films requires a deep understanding of the mechanism of sulfur incorporation into final Sb_2_(S_1-x_Se_x_)_3_ thin films. We thus first investigated the molecular species present in hydrazine-based Sb-Se, Sb-S, and Sb-S-Se solutions through Raman spectroscopy which has been proven as an effective tool in the study of hydrazine-based systems previously^18^. This characterization technique offers the unique opportunity to simultaneously probe the vibrational modes of a variety of solvated species, and consequently, we successfully uncovered the incorporation mechanism of sulfur into the Sb_2_(S_1-x_Se_x_)_3_ thin films. Through careful X-ray diffraction (XRD), energy dispersive spectroscopy (EDS) and UV-vis-NIR transmission spectroscopy, direct correlation between band gap and composition of Sb_2_(S_1-x_Se_x_)_3_ thin films across the entire compositional range from x = 0 to 1 was obtained, yielding a bowing factor of 0.118 eV. Our study not only presented a detailed chemistry picture of Sb dissovling into hydrazine, but also provided a reliable solution to conveniently tune the band gap of Sb_2_(S_1-x_Se_x_)_3_ thin films, which should find their application for antimony chalcogenide based solar cells.

## Methods

### Chemicals

Antimony powder (Sb, 99.999%), elemental sulfur (S, 99.999%), elemental selenium (Se, 99.999%), Sb_2_S_3_ powder (99.999%) and Sb_2_Se_3_ powder (99.999%) were all purchased from Alfa Aesar. Anhydrous hydrazine (N_2_H_4_, 98%) was purchased from Sinopharm Group Co. Ltd. All chemicals were used without any purification.

### Preparation of precursor solutions

All experiments were done inside a nitrogen filled glovebox (oxygen and water concentration maintained below 1 ppm). *Caution! Hydrazine is highly toxic and should be handled in the glove box with protective equipment*^19^,^20^. In order to prepare the Sb-S solutions with controlled S/Sb molar ratios ranging from 3.5 to 8, 4 mmol (0.487 g) of Sb powder combined with an appropriate amount of S were added into 2 mL hydrazine. Similarly, the Sb-Se solutions were prepared by dissolving 4 mmol of Sb powder combined with an appropriate amount of Se into 2 mL hydrazine with controlled Se/Sb molar ratios ranging from 3.5 to 6. Pure S or Se stock solution was prepared by dissolving 32 mmol elemental S or Se powder into 16 mL hydrazine and the S-Se stock solution was prepared by mixing the desired volumes of S solution and Se solution. To prepare Sb_2_(S_1-x_Se_x_)_3_ thin films, the Sb-S-Se precursor solutions were prepared by adding appropriate volumes of Se stock solution into 2 mL Sb-S solution with S/Sb molar ratio fixed as 3 and Se/(S+Se) molar ratios varied between 0.05, 0.17, 0.27 and 0.38. For example, to yield a Se/(S+Se) molar ratio equaling to 0.05 in the Sb-S-Se precursor solution, 0.3 mL Se solution (0.6 mmol Se) was added into 2 mL Sb-S solution (4 mmol Sb and 12 mmol S). Before characterization and thin film deposition, all of solutions mentioned above must be stirred for a few days until turned into homogeneous solutions with some viscosity.

### Film deposition

The Sb_2_(S_1−x_Se_x_)_3_ thin films were deposited by spin-coating Sb-S-Se precursor solutions onto 2.5 × 2.5 cm^2^ TiO_2_ substrates (the preparation method of TiO_2_ substrates has been described in detail elsewhere^4^,^21^). We used TiO_2_ substrates because of their better wettability than quartz, soda-lime glass and silicon wafer in this hydrazine-based solution process.

The Sb-S-Se precursor solutions were filtered through a 0.2 μm PTFE filter and flooded onto the substrates, then spun at 400 rpm for 10 s, followed by 2100 rpm for 45 s. After spin-coating, the films were dried on a preheated hot plate at 100 °C for 10 min, and subsequently annealed at 300 °C for 8 min.

### Materials characterization

Raman analysis was performed on the hydrazine precursor solutions including Sb-S, Sb-Se, Sb-S-Se and S-Se solutions in a backscattering confocal configuration at room temperature. A LabRAM in Via Raman system was applied for measurement, and the unpolarized light was generated through a 532 nm Ar laser with power adjusted to 50 mW. Prior to measurement, each solution was sealed in a glass vial by parafilm in order to minimize oxygen and moisture exposure. X-ray diffraction (XRD) characterization (Philips, X pert pro MRD, with Cu Kα radiation, λ = 1.54178 Å) was performed on the annealed Sb_2_(S_1-x_Se_x_)_3_ thin films prepared on TiO_2_ substrates. The compositions of the thin films were obtained through energy dispersive spectroscopy (FEI Quanta 600 scanning electron microscope, 20 kV). Sb_2_S_3_ powder (99.999%, Alfa Aesar) and Sb_2_Se_3_ powder (99.999%, Alfa Aesar) were used as standards for the calibration of EDS measurements. Scanning electron microscopy (FEI Nova NanoSEM450, without Pt coating) and UV-vis-NIR transmission spectroscopy (Perkin Elmer Instruments, Lambda 950 using integrating sphere) was performed on the Sb_2_(S_1-x_Se_x_)_3_ films to determine the surface morphology and band gap, respectively.

## Results and discussion

### Investigation of dissolution mechanism for Sb-X (X = S, Se) hydrazine precursor solutions

**Figure 1:**
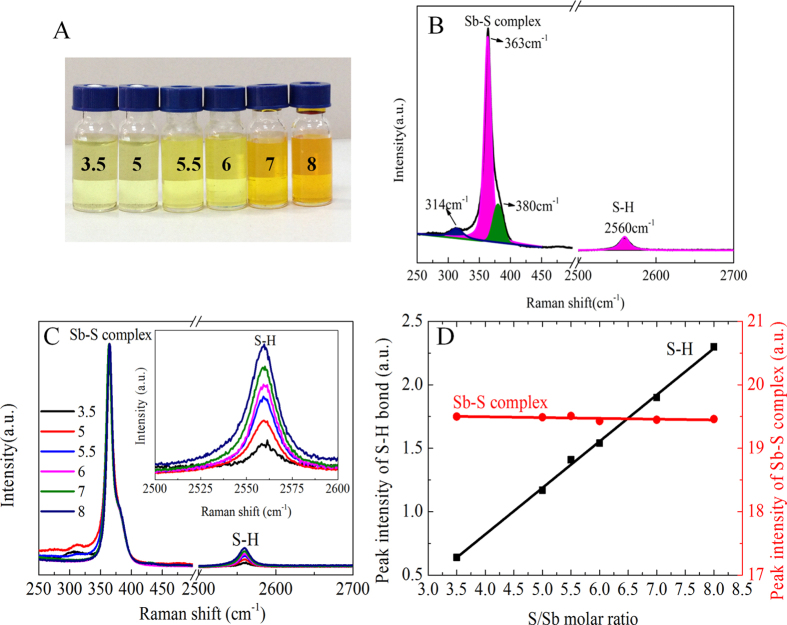
Raman spectroscopy characterization of Sb-S precursor solutions with varied S/Sb ratios. **A**) a digital picture of the Sb-S precursor solutions with varied S/Sb ratios (values marked on the vial). **B**) Multi-peak fitting of the Raman peaks obtained from the Sb-S precursor solution with an S/Sb molar ratio of 3.5. **C**) Raman spectra of Sb-S precursor solutions with different S/Sb molar ratios from 3.5 to 8. The insert was the magnified spectra of the S-H peaks at 2560 cm^−1^. **D**) The integrated intensities of the peaks for the Sb-S complex (•), and the S-H bond (■) as a function of the S/Sb molar ratio.

We first applied Raman spectroscopy to identify the molecular species presented in the Sb-S solutions prepared by dissolving Sb powder with elemental S in hydrazine. The picture of these solutions is shown in Fig. 1A, all of which are clear and transprant. Figure 1B shows the multi-peak fitting for the Raman spectrum of a 0.5 M Sb-S solution with S/Sb molar ratio of 3.5. Four distinct peaks are visible at 314, 363, 380 and 2560 cm^−1^. The first three peaks are attributable to the symmetric Sb-S stretching mode which belongs to SbS_3_ units^22^ in the Sb-S complex. The peak located at 2560 cm^−1^ is the S-H stretching mode of (N_2_H_5_)_2_S molecules formed by elemental sulfur reacted with hydrazine^23^. Figure 1C shows the Raman spectra of several Sb-S solutions, prepared with S/Sb molar ratios ranging from 3.5 to 8, and the concentration of Sb was fixed to be 0.5 M. The peaks resulting from Sb-S complex show negligible changes along with the increase of sulfur concentration; however, the S-H peaks located at 2560 cm^−1^ show increased amplitude when the S/Sb ratio gradually increased, as evidenced in the insert. This demonstrates that a certain amount of S react with Sb to form Sb-S complexes, while excess S form (N_2_H_5_)_2_S molecules in the solution. The intensities of Sb-S and S-H peaks were obtained through the multi-peak fitting and the corresponding integrated intensities were plotted as a function of the S/Sb molar ratio, as shown in Fig. 1D. Clearly, the intensity of the Sb-S peaks remained constant versus different S/Sb ratios, while the intensity of the S-H peaks increased linearly and can be traced back to its x-intercept at an S/Sb ratio of approximately 1.75. This value represents the critical point at which all of the dissolved sulfur was completely consumed by reacting with Sb and forming the Sb-S complex. We thus conclude that the S/Sb ratio of the Sb-S complex is 1.75. Therefore, the simplified chemical formula of the complex should be [Sb_4_S_7_]^2−^, which is formed through the following reaction:


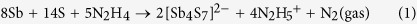


The formation of [Sb_4_S_7_]^2−^ in the Sb-S hydrazine solution agreed with previous study that [Sb_4_S_7_]^2−^ ion could steadily exsit in the alkaline aqueous solutions^22^,^24^.

**Figure 2:**
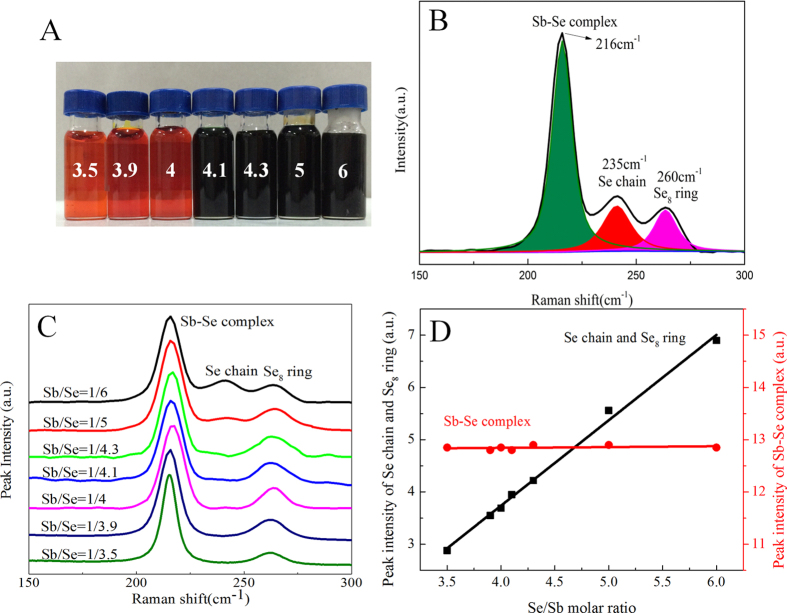
Raman spectroscopy characterization of Sb-Se precursor solutions with varied Se/Sb ratios. **A**) a digital picture of the Sb-Se precursor solutions with varied Se/Sb ratios (value marked on the vial). **B**) Multi-peak fitting of the peaks obtained from the Sb-Se precursor solution with a Se/Sb molar ratio of 6. **C**) Raman spectra of Sb-Se precursor solutions with Se/Sb molar ratios ranging from 3.5 to 6. **D**) The integrated intensities of the peaks for the Sb-Se complex (•), and the peaks for the Se chain (235 cm^−1^) and Se_8_ ring (260 cm^−1^) (■) as a function of the Se/Sb molar ratio.

Following a similar procedure, we have also analyzed the Sb-Se precursor solutions with various Se/Sb molar ratios, prepared by dissolving Sb powder with elemental Se in hydrazine. The picture of these solutions is shown in Fig. 2A. Figure 2B shows the multi-peak fitting of the spectrum of a 0.5 M Sb-Se solution with Se/Sb molar ratio of 6. Three distinct peaks are visible at 216, 235 and 260 cm^−1^, respectively. The most intense peak, located at 216 cm^−1^, can be attributed to the pyramidal SbSe_3_ stretching mode^25^ in the Sb-Se complex. The peak located at 235 cm^−1^ is assigned to Se-Se vibration in Se chains while the peak at 260 cm^−1^ represents symmetric Se-Se stretching mode in the Se_8_ rings^26^,^27^. Figure 2C shows the Raman spectra of several Sb-Se precursor solutions prepared with various Se/Sb molar ratios between 3.5 and 6. In addition to the peaks corresponding to Sb-Se complex and Se_8_ ring, some of these solutions exhibited Raman peaks corresponding to Se chain. Multi-peak fitting for each peak indicates that the integrated intensities of the peaks corresponding to Se chains and Se_8_ rings increase apparently along with the increase of Se content in the Sb-Se solution, while the integrated intensities of the Sb-Se complex peaks show negligible changes. Clearly, Se reacts with Sb forming Sb-Se complex and the leftover Se forms Se chains and/or Se_8_ rings in hydrazine. By integrating the intensities of the peaks resulting from Sb-Se complexes, Se chains and Se_8_ rings, we plotted the integrated value against the Se/Sb ratio (Fig. 2D). Obviously, the intensity of the Sb-Se peaks remained constant, while the intensity of the Se chain and Se_8_ ring increased linearly, extrapolation of the line to the x-axis produced an intercept of approximately 1.75, which is equivalently the Se/Sb ratio in the formed Sb-Se complex. Therefore, the simplified composition of Sb-Se complex present in hydrazine solutions must be [Sb_4_Se_7_]^2−^, produced from the following equation:


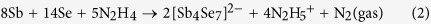


In brief, both S and Se react with Sb in hydrazine forming [Sb_4_S_7_]^2−^ and [Sb_4_Se_7_]^2−^ complexes.

### Preparation of Sb_2_(S_1-x_Se_x_)_3_ thin films

To tune the bandgap of the hydrazine processed Sb_2_(S_1-x_Se_x_)_3_ thin films, we tried to incorporate different amount of S into the final films. We prepared the precursor solutions by mixing the Sb powder, elemental S and Se altogether in hydrazine. Using a spin-coating then annealing process carried out inside the glovebox, we successfully produced the Sb_2_(S_1-x_Se_x_)_3_ (0.85 ≤ x ≤ 1) thin films (please refer to the EDS, XRD, and UV-vis-NIR transmission analysis shown in Table 1, Figure S1, and Figure S2, respectively). The general observation is that the incorporation efficiency of sulfur into the final films was very low following this strategy. For example, only when 50% sulfur was included into the precursor solution could we obtain a final Sb_2_(S_0.15_Se_0.85_)_3_ film containing 15% sulfur. Meanwhile, we tried to add more S into precursor solutions to increase the S content of final films, which was unfortunately failed due to the insolubility of the solutions. Therefore, we have to find other methods to increase the incorporation efficiency of sulfur into the final films.

**Figure 3:**
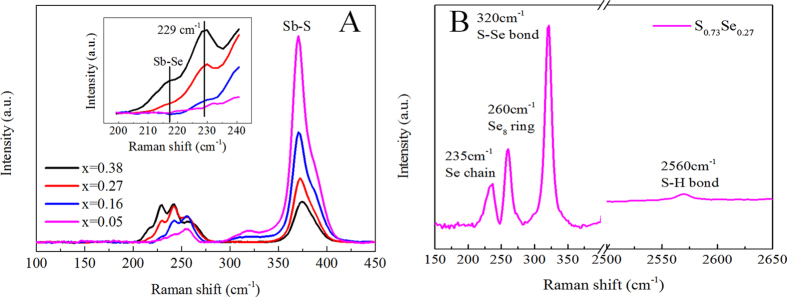
Raman spectra of Sb-S-Se precursor solutions and S_0.73_Se_0.27_ solution. **A**) Sb-S-Se precursor solutions containing different Se/(Se+S) molar ratios (0.05, 0.17, 0.27 and 0.38). The insert was the magnified spectra of the peaks located between 200 and 240 cm^−1^, B) S_0.73_Se_0.27_ solution.

In order to incorporate more S into the final Sb_2_(S_1-x_Se_x_)_3_ films, we prepared Sb-S-Se precursor solutions by adding appropriate volumes of Se stock solution into 2 mL Sb-S precursor solution prepared in advance. The preparation details were described in the experimental section. To explore the interactions between Se atoms and [Sb_4_S_7_]^2−^ complex in solution, we applied Raman spectroscopy on the Sb-S-Se solutions, which were prepared with Se/(Se+S) molar ratios ranging between 0.05 and 0.38 and stirred for at least one day before Raman characterization. As shown in Fig. 3A, the peaks located at 216 cm^−1^, 235 cm^−1^, 260 cm^−1^, and 363 cm^−1^ are attributed to [Sb_4_Se_7_]^2−^, Se chains, Se_8_ rings, and [Sb_4_S_7_]^2−^, respectively^22^,^24^,^25^,^26^,^27^. However, the molecular species corresponding to the peak at 229 cm^−1^ was unknown. To resolve this mystery, we prepared an S-Se precursor solution by mixing a desired volume of S stock solution with Se stock solution, stirred for at least one day and then measured the Raman spectrum. The S-Se solution showed four distinct peaks (Fig. 3B). Among them, the peaks at 235, 260, and 2560 cm^−1^ could be assigned to the previously observed Se chains, Se_8_ rings, and (N_2_H_5_)_2_S, while the peak at 320 cm^−1^ was ascribed to S-Se stretching mode^28^. We thus believed that the 229 cm^−1^ peak observed in the Sb-S-Se solution was attributed to Sb-S-Se species, which was probably formed by the Se substitution of S in the [Sb_4_S_7_]^2−^ complexes forming [Sb_4_S_7-x_Se_x_]^2−^ with x values depending on the amount of Se addition to the solution. Such an anion exchange is expected considering the very similar chemistry between S and Se and the unchanged complex configuration after the reaction. This result was further strengthened by comparing the intensities of [Sb_4_S_7_]^2−^ with [Sb_4_Se_7_]^2^ and [Sb_4_S_7-x_Se_x_]^2−^ in the Raman spectra. It is clear that the intensities of [Sb_4_Se_7_]^2−^ and [Sb_4_S_7-x_Se_x_]^2−^ peaks increase apparently along with the increase of Se content in the precursor solutions, while the intensity of [Sb_4_S_7_]^2−^ peaks gradually weakens. What is more, mixing Sb, S and Se atoms on a molecular level in the precursor solution facilitates the easy incorporation of S into the final films forming a homogeneous Sb_2_(S_1-x_Se_x_)_3_ alloy phase, a huge advantage for our study of alloyed Sb_2_(S_1-x_Se_x_)_3_ films.

**Figure 4:**
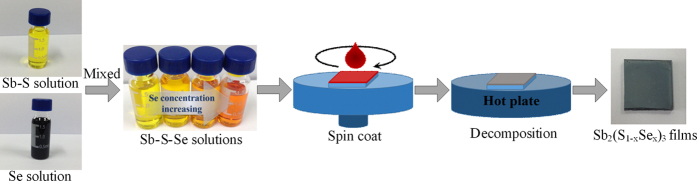
Schematic illustration of the preparation scheme of Sb_2_(S_1−x_Se_x_)_3_ thin films.

Figure 4 shows the overall preparation scheme of Sb_2_(S_1-x_Se_x_)_3_ thin films. It should be noted that we employed elemental Sb, S and Se instead of conventional metal chalcogenide^29^,^30^ to eliminate possible material composition variation from batch to batch and hence ensure precise composition control in the final film. Deposition of Sb_2_(S_1-x_Se_x_)_3_ films involved four steps: (i) dissolve Sb with S in hydrazine to produce the Sb-S stock solution and dissolve Se in hydrazine to produce Se stock solution at room temperature; (ii) prepare the Sb-S-Se precursor solutions by mixing the Sb-S solution with different volumes of Se solutions, followed by stirring for at least 2 h before spin-coating; (iii) spin-coat the Sb-S-Se solutions onto prepared TiO_2_ substrates and (iv) anneal the as-deposited films at 300 °C for 8 min to yield the Sb_2_(S_1-x_Se_x_)_3_ thin films. In addition, the binary Sb_2_S_3_ and Sb_2_Se_3_ films were also prepared using the Sb-S (S/Sb = 3) and Sb-Se (Se/Sb = 3) solutions with the same annealing process.

**Figure 5:**
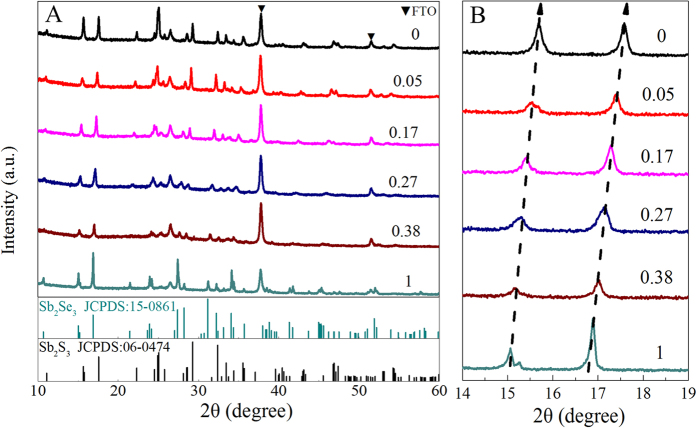
Phase characterization of Sb_2_(S_1−x_Se_x_)_3_ (0 ≤ x ≤ 1) alloy films with different Se concentration. **A**) XRD patterns of the Sb_2_(S_1-x_Se_x_)_3_ alloy films with their Se concentration in the Sb-S-Se precursor solutions indicated; **B**) enlarged (020) and (120) XRD peaks of the same films as in panel A.

Se content (x) in the Sb_2_(S_1-x_Se_x_)_3_ films could be tuned across the entire range from 0 to 1 by varying the volumes of Se stock solution added into Sb-S solution. X-ray diffraction (XRD) was employed to characterize the formation of Sb_2_(S_1-x_Se_x_)_3_ alloy phase. Figure 5A shows the XRD patterns of six different Sb_2_(S_1-x_Se_x_)_3_ films produced using Sb-S-Se precursor solutions with Se/S+Se molar ratios of 1, 0.38, 0.27, 0.17, 0.05, and 0, respectively. The variation of the Se/(S+Se) ratio in the Sb_2_(S_1-x_Se_x_)_3_ films is clearly reflected from the XRD patterns. At x = 1, the XRD pattern corresponds to orthorombic Sb_2_Se_3_ (JCPDS no. 15-0861). As the sulfur concentration increases (that is, x decreases), the diffraction peaks gradually shift to larger 2θ angle which is clearly visible in the magnified view of (020) and (120) XRD peaks shown in Fig. 5B. Such a shift to higher angles is attributed to the drcreased lattice constants of the films with gradul substitution of larger Se atoms (1.98 Å) with smaller S atoms (1.84 Å). At x = 0, the XRD pattern could be well indexed to orthorombic Sb_2_S_3_ (JCPDS no. 06-0474). It should be emphasized that none of our samples have any peaks corresponding to other phases (except from the peaks of FTO substrate labelled with ▼), indicating that these samples are single-phase, forming the Sb_2_(S_1−x_Se_x_)_3_ alloy films.

**Figure 6:**
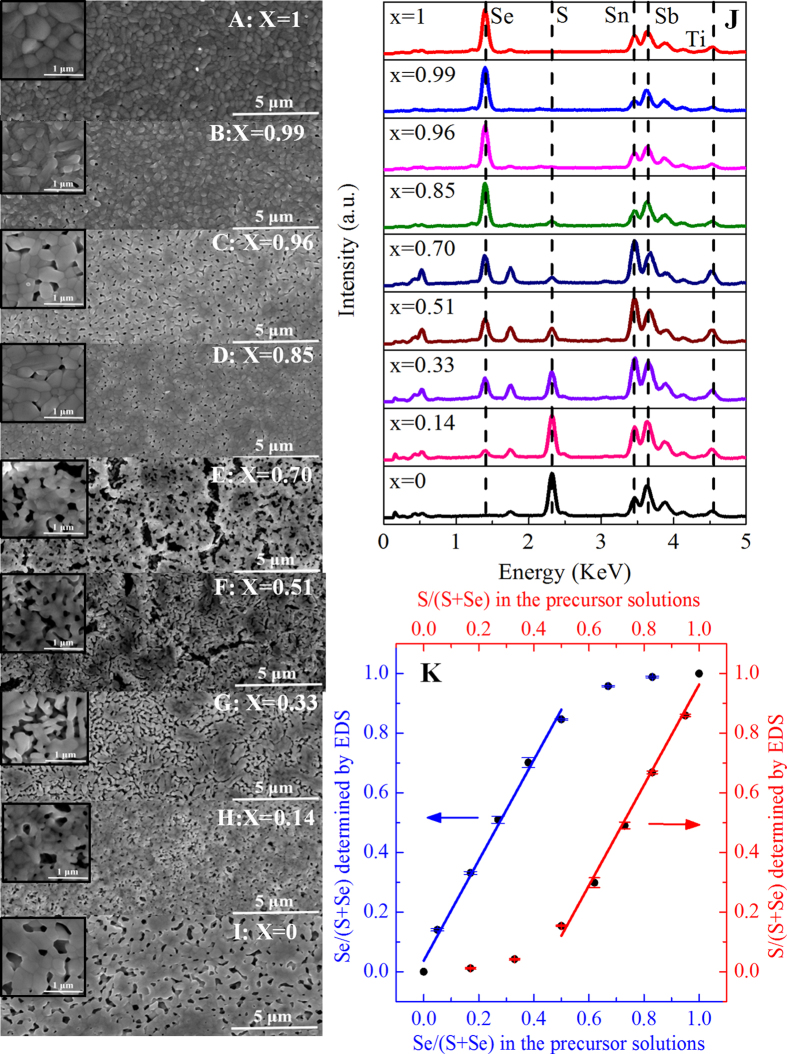
Morphology and composition characterization of Sb_2_(S_1−x_Se_x_)_3_ (0 ≤ x ≤ 1) alloy films with different Se concentration. (**A-I**) Top view SEM images of Sb_2_(S_1−x_Se_x_)_3_ (0 ≤ x ≤ 1) alloy films marked with their Se concentration x of 1, 0.99, 0.96, 0.85, 0.70, 0.51, 0.33, 0.14, and 0, respectively. Insets show the enlarged images of the corresponding alloy films. (**J**) EDS patterns of Sb_2_(S_1-x_Se_x_)_3_ (0 ≤ x ≤ 1) alloy films; Ti and Sn signals are from TiO_2_ and FTO substrates, respectively. (**K**) Plot of Se concerntration x in the final films calculated from the EDS spectra versus Se concentration in the Sb-S-Se precursor solutions; similarly, S concerntration 1-x in the final films calculated from from the EDS spectra plotted versus the S concentration in Sb-S-Se precursor solutions. The error bars represent the uncertainties of the instrument measurements.

Scanning electron microscopy (SEM) was applied to characterize the morphologies of Sb_2_(S_1−x_Se_x_)_3_ films (Fig. 6A–I) marked with Se concentration *x* of 1, 0.99, 0.96, 0.85, 0.70, 0.51, 0.33, 0.14, and 0, respectively. The high magnification SEM images shown in the insets indicated that the grain size of Sb_2_(S_1−x_Se_x_)_3_ thin films varied from 200 to 900 nm. Apparently, more cracks and pinholes appeared in the final films as the sulfur content increased in the Sb_2_(S_1−x_Se_x_)_3_ alloy films, which may result from the easier volatilization of sulfur and hence larger volume shrinkage and more violent volume change during the annealing of Sb_2_(S_1−x_Se_x_)_3_ films. The chemical compositions of Sb_2_(S_1−x_Se_x_)_3_ films were analyzed by EDS as shown in Fig. 6J. A quantitative elemental EDS analysis of these films revealed that the measured Se concentration x for each sample was 1.0, 0.99, 0.96, 0.85, 0.70, 0.51, 0.33, 0.14, and 0, which were listed in Table 1. It should be noted that our EDS facility was calibrated using commericial Sb_2_S_3_ and Sb_2_Se_3_ powder (Alfa Aesar, 99.999% pure) in advance; and for the composition calculation, EDS results from three arbitrarily selected spots of each sample were averaged to obtain the final results. The relationship between the Se or S content in the precursor solutions and those in Sb_2_(S_1−x_Se_x_)_3_ films gave a non-linear trend, as shown in Fig. 6K. For Se, the content in Sb_2_(S_1−x_Se_x_)_3_ films showed first a superlinearly and then a slowly increasing trend with increased Se content in the precursor solutions, and the demarcation point was 0.5. On the contrary, S content in the resulting Sb_2_(S_1−x_Se_x_)_3_ films showed first a slowly (sub-linear) followed by a linearly increasing trend with increased S content in the precursor solutions, and the demarcation point was 0.5 as well. Clearly, Se was more competitive in being incoporated into the Sb_2_(S_1−x_Se_x_)_3_ films than S. In brief, with the goal to increase the S content of the final films, the approach of adding Se solution into Sb-S solution was more effective than that of mixing the Sb powder, elemental S and Se altogether in hydrazine.

**Figure 7:**
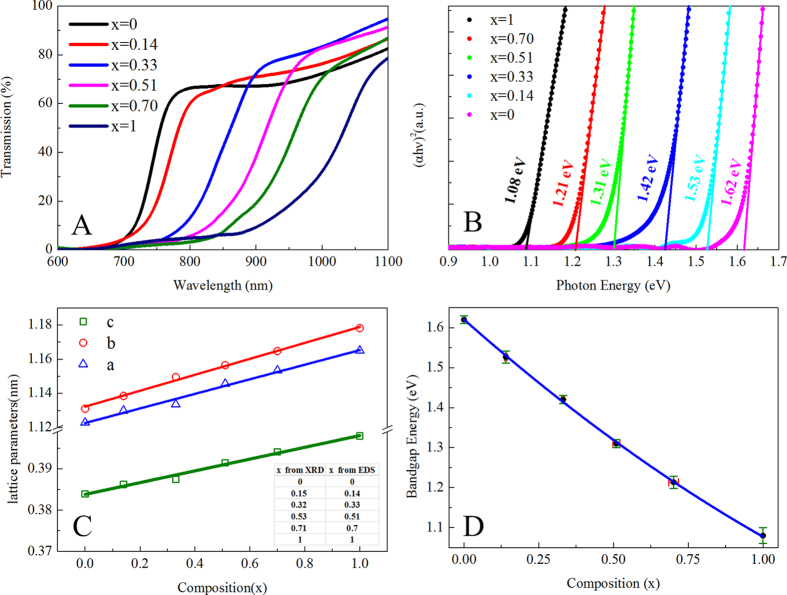
Optical properties of Sb_2_(S_1−x_Se_x_)_3_ (0 ≤ x ≤ 1) alloy films with different Se concentration. **A**) UV-vis-NIR transmission spectra of Sb_2_(S_1−x_Se_x_)_3_ (0 ≤ x ≤ 1) alloy films; **B**) Pots of (*αhv*)^2^ vs the photon energy (*hv*) reveal the band-gaps of Sb_2_(S_1−x_Se_x_)_3_ alloy films as 1.08, 1.21, 1.31, 1.42, 1.53, and 1.62 eV for x = 1, 0.70, 0.51, 0.33, 0.14, and 0, respectively; **C**) Lattice constants a (blue), b (red), and c (green), derived from XRD diffraction peaks, plotted as functions of Se concentration x in the Sb_2_(S_1-x_Se_x_)_3_ alloy films. The inserted table shows the comparison of x derived from XRD and EDS. **D**) The band gap energy plotted verus the Se concentration x in the Sb_2_(S_1-x_Se_x_)_3_ alloy films. The solid curve is a quadratic fit to the measured values of the band gap energy. The error bars labled by the red and green colors represent the uncertainties in the determination of the composition and the optical band gap of alloy films, respectively.

Analysis of optical transmission spectra is one of the most effective tools for understanding the band structure and energy gaps of semiconductor materials. As shown in the Fig. 7A, optical transmission spectra of the Sb_2_(S_1-x_Se_x_)_3_ films exhibite a gradual red shift toward longer wavelength with increased Se composition, which is due to a narrower band gap of Sb_2_Se_3_ (~1.08 eV) than that of Sb_2_S_3_ (~1.62 eV). For semiconductors, the optical absorption near band edge follows the formula: (αhν)^n^ = A^n^(hν−E_g_), where A is a constant, h is the Planck’s constant, ν is the frequency of the incident photon, E_g_ is the band gap and n equals to 2 for direct band gap semiconductors and to 1/2 for an indirect band gap semiconductor^31^. Although the optical transition type of Sb_2_Se_3_ and Sb_2_S_3_ was still controversial^32^, analysis assuming direct band gap yielded a high quality fitting ((αhν)^2^ versus the photon energy (hν), Fig. 7B) while fitting assuming indrect band gap failed. The estimated mean band gaps for our samples were found to be Eg = 1.62, 1.53, 1.42, 1.31, 1.21, and 1.08 eV, as listed in Table 1. The E_g_ was thus seen to increase from 1.08 eV for Sb_2_Se_3_ to 1.62 eV for Sb_2_S_3_ as the concentration of S increases in the Sb_2_(S_1-x_Se_x_)_3_ series.

For verifying the composition more precisely, we also determined the Se concentration using XRD patterns. Figure 7C shows the lattice parameters of a, b and c, determined from XRD^33^ (a, b and c are derived from the (200), (020) and (221) peaks respectively), plotted as a function of the Se concentration x in the Sb_2_(S_1−x_Se_x_)_3_ alloy films. The Se concentration x was determined by averaging the values obtained from calibrated and repeated EDS measurements (first calibrated the system using commercial 99.999% Sb_2_S_3_ and Sb_2_Se_3_ powder and then measured the EDS value over 6 arbitrarily selected points in two rounds). As the figure shows, the experimentally measured a, b and c values could be well fitted by a straight line, which indicated that the dependence of lattice parameters on alloy composition conforms with the Vegard’s law approximation^34^:


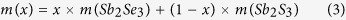


where *x*, m(*x*), m(Sb_2_Se_3_) and m(Sb_2_S_3_) are the respective Se concentration, lattice parameters *a*, *b*, and *c* of these orthorhombic structured samples. According to formula (3), the Se concentration *x* was determined, as listed in the inserted table, which is very close to the results obtained from EDS, further confirming the composition of the alloyed Sb_2_(S_1−x_Se_x_)_3_ films. To further understand the relation between the alloy composition and their band gap evolution, Fig. 7D plots the band gap of Sb_2_(S_1−x_Se_x_)_3_ films as a function of Se concentration x. The band gap was also obtained by multi-measurements on different films. It is found that the optical band gap of alloy samples shows a curve, which is termed as band gap “bowing”^35^. The data points are fitted by the modified bowing equation as follows:


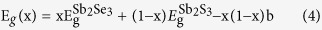


where 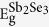 and 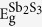 are band gap energies of pure Sb_2_Se_3_ and Sb_2_S_3_, respectively; b is the bowing parameter describing the nonlinear relationship between band gap and Se concentration x. Through fitting by the quadratic equation mentioned above, the dependence of the optical band gap on Se concentration x is characterized by the following relation:


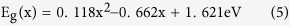


The bowing parameter of Sb_2_(S_1-x_Se_x_)_3_ alloy films is determined as 0.118 eV. Such a nonlinear dependence of band gap on Se concentration x could possibly arise from three souces: (i) the change of band structure due to lattice constant variation, (ii) electron distribution deformation due to electronegativity differences of the alloy atoms, and (iii) relaxation of anion-cation bond lengths and angles^36^,^37^. Recent reports of amorphous thin films of Sb_2_(S_1−x_Se_x_)_3_ (x = 0, 1/3, 2/3, and 1) solid solutions^9^ and Sb_2_(S_1-x_Se_x_)_3_ (0 ≤ x ≤ 1) nanotubes^10^ indicate quadratically increasing optical band gap energy with sulfur content, which is in good agreement with our findings. The bowing parameter “b” reported in these two papers are 0.02 and 0.03 eV, respectively, which are smaller than our 0.118 eV. This may results from the difference of preparation process. On account of the extremely careful composition (EDS and XRD) and band gap measurements, we believe that our results are credible for polycrystalline Sb_2_(S_1−x_Se_x_)_3_ (0 ≤ x ≤ 1) alloy films, which is more relevant for photovoltaic application. We summarize our experimental results on the composition and band gap study of hydrazine solution processed Sb_2_(S_1−x_Se_x_)_3_ films in Table 1.

## Conclusions

In summary, we have systematically studied the dissolution mechanism of Sb in hydrazine, and the composition dependent band gap of Sb_2_(S_1−x_Se_x_)_3_ (0 ≤ x ≤ 1) alloy films. The major findings are: 1) Sb reacts with S and Se in hydrazine forming (N_2_H_5_)_2_Sb_4_S_7_ and (N_2_H_5_)_2_Sb_4_Se_7_ molecules producing clear solutions; 2) mixing of Se hydrazine solution with Sb-S hydrazine solution leads to anion mixing on a molecular level, facilitating production of highly uniform polycrystalline Sb_2_(S_1−x_Se_x_)_3_ thin films at a relatively low temperature; 3) for Sb_2_(S_1−x_Se_x_)_3_ alloy films, the XRD peaks’ position dependence on composition is linear while the the band gap dependence on composition is quadratical and it follows the equation 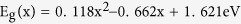, where the bowing parameter is 0.118 eV. Our study provide a valuable guidance for the band gap tunning of Sb_2_(S_1−x_Se_x_)_3_ (0 ≤ x ≤ 1) alloy films, which should find their application for antimony chalcogenide based solar cells.

## Additional Information

**How to cite this article**: Yang, B. *et al*. Hydrazine solution processed Sb_2_S_3_, Sb_2_Se_3_ and Sb_2_(S_1–x_Se_x_)_3_ film: molecular precursor identification, film fabrication and band gap tuning. *Sci. Rep*. **5**, 10978; doi: 10.1038/srep10978 (2015).

## Supplementary Material

Supplementary Information

## Figures and Tables

**Table 1:** Results summary for the composition and optical band gap energies of hydrazine processed Sb_2_(S_1-x_Se_x_)_3_ films.

Target composition Sb_2_(S_1-x_Se_x_)_3_	Precursor composition (Sb/S/Se atom ratio %)	Composition measured by EDS^a^ (Sb/S/Se atom ratio %)	Alloy film stoichiometry	E_g_ (eV)^b^
Sb_2_S_3_	25:75:0 (2:6:0)	39.14:60.86:0	Sb_1.96_S_3.04_ (x = 0)	1.62
Sb_2_(S_0.95_Se_0.05_)_3_	24.1:72.29:3.61 (2:6:0.31)	38.24:53.11:8.65	Sb_1.91_S_2.66_Se_0.43_(x = 0.14)	1.53
Sb_2_(S_0.83_Se_0.17_)_3_	21.74:65.22:13.02 (2:6:1.2)	39.65:40.43:19.92	Sb_1.98_S_2.02_Se_1_(x = 0.33)	1.42
Sb_2_(S_0.73_Se_0.27_)_3_	19.51:58.54:21.95 (2:6:2.25)	42.21:28.32:29.47	Sb_2.12_S_1.42_Se_1.46_(x = 0.51)	1.31
Sb_2_(S_0.62_Se_0.38_)_3_	17.24:51.72:31.04 (2:6:3.6)	42.28:17.32:40.39	Sb_2.12_S_0.86_Se_2.02_(x = 0.70)	1.21
Sb_2_(S_0.50_Se_0.50_)_3_	25:37.5:37.5 (2:3:3)	38.34:9.48:52.18	Sb_1.92_S_0.47_Se_2.61_(x = 0.85)	1.20
Sb_2_(S_0.33_Se_0.67_)_3_	25:25:50 (2:2:4)	39.1:2.52:58.38	Sb_1.96_S_0.13_Se_2.91_(x = 0.96)	1.11
Sb_2_(S_0.17_Se_0.83_)_3_	25:12.5:62.5 (2:1:5)	41.24:0.59:58.17	Sb_2.01_S_0.03_Se_2.96_(x = 0.99)	1.12
Sb_2_Se_3_ (x = 1)	25:0:75 (2:0:6)	39.20:0:60.80	Sb_1.96_Se_3.044_(x = 1)	1.08

^a^EDS measurements have an error which represents the uncertainty of the instrument measurements.

^b^Optical band gap energies (E_g_) were determined from UV-vis-NIR spectra.
