# Supplementary material for: Hydrazine solution processed Sb2S3, Sb2Se3 and Sb2(S1−xSex)3 film: molecular precursor identification, film fabrication and band gap tuning
Source: Sci Rep. 2015 Jun 4;5:10978. doi: 10.1038/srep10978 (PMC4455288; doi:10.1038/srep10978)
Supplement: Supplementary Information [file srep10978-s1.pdf]

Supplementary Information

Hydrazine solution processed Sb_2S_3, Sb_2Se_3 and $Sb_2(S_{1-x}Se_x)_3$ film: molecular precursor identification, film fabrication and band gap tuning

Bo Yang[1,2+], Ding-Jiang Xue[1,2+], Meiying Leng[1,2], Jie Zhong[1,2], Liang Wang[1,2], Huaibing Song[1,2], Ying Zhou[1,2], Jiang Tang[1,2*]

[1]Wuhan National Laboratory for Optoelectronics (WNLO), and [2] School of Optical and Electronic Information, Huazhong University of Science and Technology (HUST), Wuhan, 430074, China

[+]These authors contribute equally to this work.

*Correspondence and requests for materials should be addressed to J. T. (E-mail: jtang@mail.hust.edu.cn)

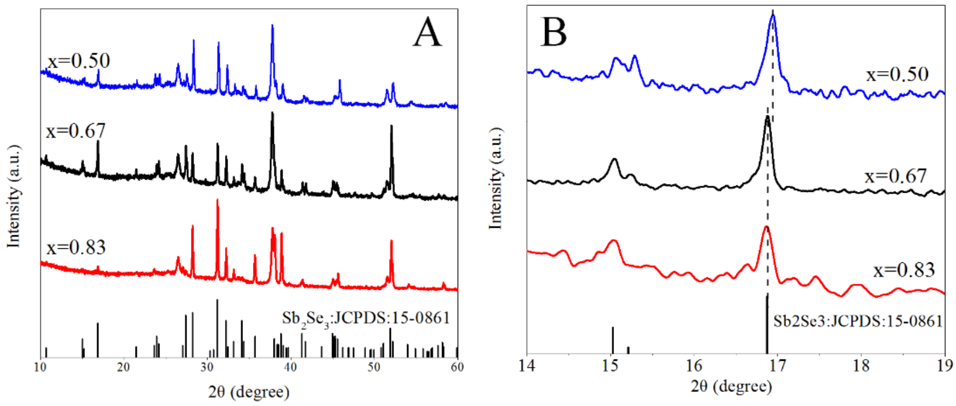

Figure S1 | A) From bottom to top, XRD patterns of the $Sb_2(S_{1-x}Se_x)_3$ (0≤x≤1) alloy films with their with their Se concentration in the precursor solutions indicated; B) (020) and (120) XRD peaks of the same films as in panel A.

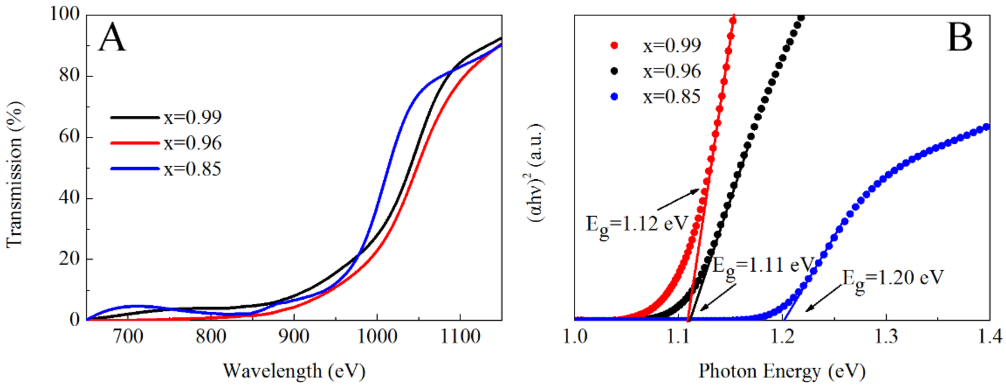

Figure S2 | A) UV-vis-NIR transmission spectra of $Sb_2(S_{1-x}Se_x)_3$ (0≤x≤1) alloy films marked with their selenium concentration x of 0.99, 0.96, and 0.85, respectively. All of the samples were

annealed at 300 ℃ for 8 min. B) Pots of $(\alpha h\nu)^2$ vs the photon energy ($h\nu$) reveal the band-gaps of $Sb_2(S_{1-x}Se_x)_3$ ($0 \leqslant x \leqslant 1$) alloy films as 1.12, 1.11, and 1.10 eV for x = 0.99, 0.96, and 0.85, respectively;
